# More Than a Headache: Unveiling Pituitary Apoplexy Following Acute Coronary Syndrome

**DOI:** 10.7759/cureus.96188

**Published:** 2025-11-06

**Authors:** Gisela Gonçalves, Daniela R Alves, Ana Oliveira, Bruna Nascimento, Andreia Lopes

**Affiliations:** 1 Internal Medicine, Centro Hospitalar do Baixo Vouga, Aveiro, PRT; 2 Internal Medicine, Unidade Local de Saúde da Região de Aveiro, Aveiro, PRT

**Keywords:** acute coronary syndrome, anticoagulation, endocrine emergency, pituitary adenoma, pituitary apoplexy

## Abstract

Pituitary apoplexy (PA) is an endocrine emergency characterized by ischemia or hemorrhage of the pituitary gland. The main symptom is a sudden and severe headache, often accompanied by ocular palsies and visual field defects. Recognized risk factors include anticoagulation, hypertension, obesity, and major surgical procedures. A 72-year-old man with a history of partially resected pituitary macroadenoma was admitted for an acute coronary syndrome. Anticoagulant and antiplatelet therapy were initiated, and he was awaiting coronary artery bypass grafting (CABG). He experienced a sudden headache accompanied by palsies of the left third and sixth cranial nerves. An initial computed tomography (CT) scan revealed no remarkable findings; however, due to high clinical suspicion of PA, hydrocortisone treatment was initiated, and anticoagulation therapy was discontinued. No new hormonal deficits were observed. Subsequently, magnetic resonance imaging (MRI) confirmed the diagnosis. Following a multidisciplinary consultation, a conservative management strategy was adopted. The patient's symptoms gradually subsided, and he successfully underwent CABG. This case highlights the limited sensitivity of CT scan, emphasizing MRI as the gold standard for diagnosis. The patient's acute coronary syndrome likely triggered the event, not only due to the antithrombotic therapy used but also through shared risk factors such as obesity. Although many patients require surgical intervention, certain cases may be managed effectively through conservative measures. This case demonstrates a successful outcome with a conservative approach, a strategy suitable for clinically stable patients who do not have severe or worsening visual deficits.

## Introduction

Pituitary apoplexy (PA) is a life-threatening condition characterized by infarction and/or hemorrhage of the pituitary gland. In approximately 80% of cases, PA constitutes the first manifestation of an underlying pituitary tumor [1], and this occurrence is more prevalent in macroadenomas. It occurs more frequently in males (66.8%), with the highest incidence observed between the fifth and sixth decades of life [2]. Enlargement of the pituitary gland results in increased intrasellar pressure, which subsequently causes mechanical compression of adjacent neurovascular structures, particularly the optic tracts and cavernous sinus.

The clinical spectrum ranges from subclinical or minimally symptomatic cases to an acute presentation characterized by severe neurological deficits, with an increased mortality risk. A thunderclap headache is identified as the main symptom, frequently accompanied by nausea and vomiting [2,3]. Typically, the headache originates in the retro-orbital or frontal regions. It may initially present unilaterally and subsequently become generalized. Significant neuro-ophthalmologic signs include diplopia, ptosis, visual field defects (more frequently bitemporal hemianopsia), and diminished visual acuity [2,3]. Ophthalmoplegia results from lateral compression of the cavernous sinus, impacting the cranial nerves (CN) that traverse this region. The oculomotor nerve (CN III) is most frequently affected, followed by the trochlear nerve (CN IV) and the abducens nerve (CN VI). Clinical manifestations consistent with hypopituitarism and hyponatremia are commonly observed [3]. In some instances, hypophyseal hemorrhage may extend into the subarachnoid space, leading to meningismus and vasospasm, which can subsequently result in cerebral infarcts [4]. Precipitating factors are identified in up to 40% of patients [3,5]. Certain cardiovascular risk factors, such as arterial hypertension and obesity, are prevalent [3]. Additional factors include major surgical procedures, such as coronary bypass, anticoagulant therapy or coagulopathies, pregnancy, severe arterial hypotension, radiotherapy, and pituitary stimulation tests with gonadotropin and corticotropin-releasing hormones [5,6].

The association between PA and acute cardiovascular events, including acute coronary syndrome (ACS), is of considerable significance due to the presence of multiple, interacting risk factors. First, patients with ACS often exhibit pre-existing risk factors for PA, such as arterial hypertension, diabetes mellitus, and obesity, which may contribute to underlying endothelial dysfunction and vascular fragility of the adenoma [2,3]. Second, the ACS event itself induces significant physiological stress and may cause hemodynamic instability, potentially compromising the fragile blood supply to the pituitary gland and leading to ischemia. Lastly, and most commonly, PA can be precipitated by antithrombotic therapies, including antiplatelet agents and anticoagulants, both employed in ACS management. These medications can trigger significant bleeding within the tumor's abnormal vasculature [6].

The diagnosis is established based on the clinical presentation and confirmed through imaging tests, preferably magnetic resonance imaging. Treatment is tailored to each patient and encompasses both medical and surgical interventions. Due to its complexity and severity, managing PA requires a multidisciplinary team comprising endocrinologists, internists, neuroradiologists, neurosurgeons, and ophthalmologists.

## Case presentation

We present a case of a 72-year-old male with multiple cardiovascular risk factors, including obesity and dyslipidemia, as well as a significant neuro-endocrine history of a pituitary macroadenoma (partially resected in 2008 and 2016, resulting in isolated gonadotropin deficiency treated with testosterone supplementation). He was admitted for a non-ST-elevation myocardial infarction and initially treated with ticagrelor, aspirin, and low molecular weight heparin. Following coronary angiography, which confirmed three-vessel disease, ticagrelor was discontinued, and the patient was maintained on low molecular weight heparin and aspirin while undergoing preoperative workup for coronary artery bypass grafting (CABG).

**Figure 1:**
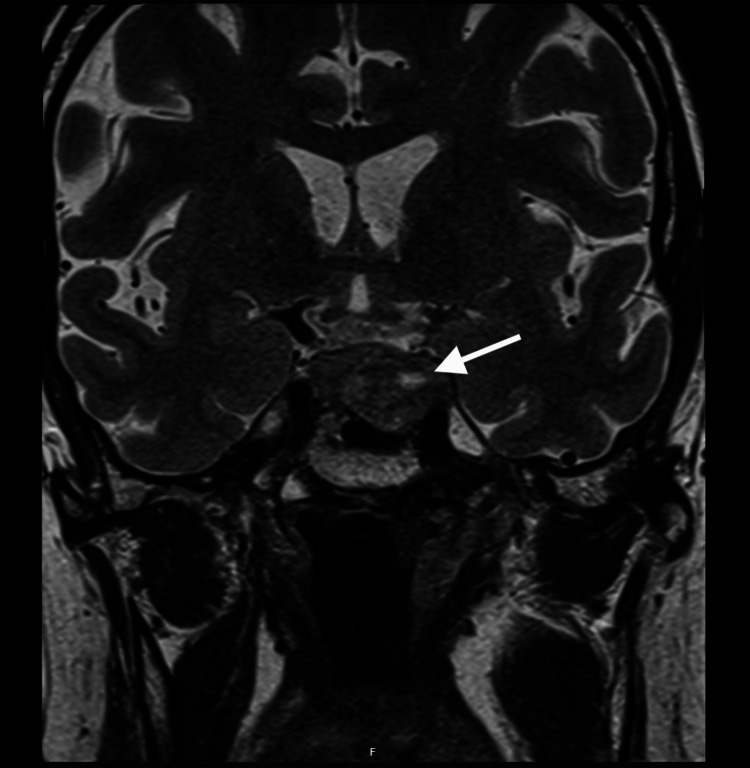
T2-weighted magnetic resonance imaging The image reveals an enlarged adenoma with a heterogeneous appearance (white arrow), containing mixed areas of hyperintensity and hypointensity suggestive of intralesional hemorrhage and/or necrosis.

On the seventh day of admission, the patient first developed a mild frontal headache, which was initially responsive to standard analgesics. The following day, the headache intensified and was accompanied by nausea. Neuro-ophthalmological examination revealed complete left-sided ptosis and mydriasis, with an absent direct pupillary light reflex. Furthermore, the left eye demonstrated limited abduction, resulting in horizontal diplopia most evident on leftward gaze. These findings were collectively indicative of a left third and sixth cranial nerve palsy. No additional neurological deficits were observed, nor were there signs suggestive of acute hypopituitarism. An urgent cerebral computed tomography (CT) angiography was conducted, revealing no evidence of an acute vascular event and ruling out large vessel occlusion. Although the CT confirmed the pre-existing intrasellar mass, it showed no clear evidence of acute hemorrhage or ischemia. Considering the heightened clinical suspicion of PA - attributable to severe headache and new cranial nerve deficits within the context of antithrombotic therapy in a patient with a known pituitary macroadenoma - hydrocortisone treatment was initiated at a dosage of 100 mg every six hours. The anticoagulation therapy was promptly discontinued, maintaining the patient solely on aspirin. Laboratory results confirmed stable sodium and coagulation parameters, with the pituitary function testing indicating no new hormonal deficiencies (Table 1). An ophthalmological evaluation confirmed preserved visual acuity and visual fields. Magnetic resonance imaging (MRI) subsequently confirmed the clinical diagnosis, revealing significant enlargement and a heterogeneous intrasellar mass with clear evidence of intralesional necrosis and hemorrhage (Figure 1). Given the patient's stability, preserved vision, and the absence of worsening neuro-ophthalmological deficits, a decision was reached after multidisciplinary team consultation to pursue a conservative management strategy with gradually tapering hydrocortisone and meticulously monitoring clinical and laboratory parameters. The patient remained clinically stable, the headache completely resolved, and the ophthalmoparesis gradually improved.

**Table 1:** Laboratory results ACTH: adrenocorticotropin; free T4: serum free thyroxine; FSH: follicle-stimulating hormone; IGF-1: insulin-like growth factor 1; LH: luteinising hormone; TSH: thyroid-stimulating hormone

	Results	Reference values
TSH	1.4mU/L	0.55-4.78mU/L
Free T4	1.25ng/dL	0.80-1.80ng/dL
Prolactin	5.5ng/mL	2.1-17.7ng/mL
ACTH	15.0pg/mL	7.2-63.3pg/mL
Cortisol	14.79µg/dL	4.82-19.50µg/dL
LH	2.63mUI/mL	0,57-12.07mUI/mL
FSH	5.34 mUI/mL	0,95-11.95mUI/mL
Testosterone	2.17ng/mL	1,93-7,40ng/mL
IGF-1	57µg/L	50-195µg/L
Sodium	134mEq/L	132-146mEq/L

He underwent CABG on the thirty-fifth day of admission under stress-dose hydrocortisone coverage, with no complications. At discharge, he exhibited complete resolution of symptoms and no new hormonal deficits. A gradual tapering of corticosteroid therapy was maintained. A follow-up MRI demonstrated reabsorption of the hemorrhage and necrotic areas, along with a reduction in the adenoma size. Consequently, the patient continues to be monitored regularly by a dedicated pituitary tumors team.

## Discussion

Most PA cases occur in pre-existing pituitary adenomas, whose abnormal and fragile blood vessels are inherently vulnerable to ischemic or hemorrhagic events. However, it can also develop in healthy pituitary glands. Diagnosing PA can be challenging due to its symptoms often mirroring those of more prevalent neurological emergencies. If a patient presents with an intense, sudden headache accompanied by neuro-ophthalmologic symptoms, PA should always be considered, and an immediate, multidisciplinary assessment is crucial.

A CT scan is generally employed as the initial imaging modality due to its rapid acquisition and widespread availability within emergency settings. However, as demonstrated in our case, where the initial CT only revealed the pre-existing sellar mass without acute hemorrhage or ischemia, its sensitivity for detecting early signs of PA is limited [7]. A CT scan is also advantageous in ruling out other differential diagnoses with similar clinical presentations such as subarachnoid hemorrhage [7]. Cerebral MRI is considered the gold standard imaging modality owing to its sensitivity of approximately 90% [8]. The superior soft-tissue resolution of MRI and its capacity to detect blood products at various stages [8] were crucial in confirming the clinical diagnosis of intralesional hemorrhage and necrosis in our patient. This substantiates the recommendation that cerebral MRI should be performed in all patients exhibiting a clinical suspicion of PA, even when the CT findings are non-suggestive [8].

Although there is no conclusive evidence indicating an increased risk of PA in patients experiencing ACS, there appears to be a relationship between PA and pre-existing cardiovascular events [2]. It is essential to consider similar risk factors such as hypertension and obesity [2]. These factors contribute to endothelial dysfunction [2] and may be present alongside medication used in ACS. Furthermore, a significant cardiac event induces a widespread stress response, which may alter blood flow and pressure within the abnormal adenoma vasculature, thereby increasing the risk of ischemia or vessel rupture with hemorrhage. Identifying all potential risk factors for PA is imperative, facilitating their elimination whenever feasible.

Our patient presented a considerable management challenge due to the concurrent need for antithrombotic therapy for ACS and the potential risk of exacerbating pituitary hemorrhage. Approximately 16-36% of patients with PA have been reported to be using anticoagulant or antiplatelet medication [6]. Both are employed in managing ACS, and our patient was initially treated with both pharmacological classes. Given the conflicting priorities, the anticoagulation therapy was promptly discontinued upon high clinical suspicion of PA. The decision to maintain aspirin alone was a delicate, multidisciplinary choice intended to reduce the risk of pituitary hemorrhage while ensuring minimal necessary antithrombotic coverage to mitigate the risk of recurrent ischemic events. This decision highlights the need for immediate, individualized risk stratification in similar settings, as there is currently no conclusive evidence concerning the optimal antithrombotic strategy to employ.

The optimal PA management remains a matter of debate, particularly concerning which patients require surgical treatment and the appropriate timing for surgical intervention. The patient's neurological status should be the primary determinant in managing PA. While up to two-thirds of cases may require urgent surgical intervention [9] - recommended for severe or progressively worsening neuro-ophthalmologic deficits, particularly visual acuity or field loss, or a decline in consciousness - medical treatment with high-dose corticosteroids is essential for all patients. Medical treatment additionally encompasses managing fluid and electrolytes, addressing hormonal deficiencies, and stabilizing hemodynamics when necessary. In our case, a conservative management approach was justified because the patient showed maintained visual acuity and fields, stability, and signs of improvement in the non-progressive cranial nerve palsies. The excellent outcome, confirmed by the complete resolution of ophthalmoparesis and the favorable follow-up MRI indicating hemorrhage reabsorption, validates this patient-centered strategy and reinforces the existing literature supporting conservative management in carefully selected, clinically stable cases.

## Conclusions

In conclusion, this case report details a complex presentation of PA following ACS in a patient with a known macroadenoma. It highlights several crucial clinical lessons. First, it emphasizes the diagnostic challenge of PA and underscores the importance of maintaining a high level of clinical suspicion. Even when initial CT scans reveal no signs of PA, MRI should be pursued as the definitive imaging modality. Second, it illustrates the intricate interplay of risk factors, whereby a significant cardiovascular event and its necessary treatment may precipitate a neuroendocrine emergency. This presents a critical management challenge, as the potential for deteriorating PA must be promptly balanced against the necessity of administering antithrombotic therapy for ACS. Accordingly, the management of PA must be individualized and necessitates an immediate, patient-centered, multidisciplinary strategy.

A conservative approach was successfully employed in this case. The excellent outcome, demonstrated by the complete resolution of ophthalmoparesis and a favorable follow-up MRI, validates this patient-centric approach and advocates for conservative management in carefully selected cases.
